# Combined oral prednisolone and heparin versus heparin: the effect on peripheral NK cells and clinical outcome in patients with unexplained recurrent miscarriage. A double-blind placebo randomized controlled trial

**DOI:** 10.1007/s00404-014-3262-0

**Published:** 2014-05-13

**Authors:** Mostafa F. Gomaa, Abdellatif G. Elkholy, Mourrad M. El-Said, Nermine E. Abdel-Salam

**Affiliations:** Department of Obstetrics and Gynecology, Faculty of Medicine, Ain Shams University, Egypt 28 Aly Ameen Street, Naser City, Cairo, Egypt

**Keywords:** Recurrent miscarriage, Natural killer cells, Prednisolone, Heparin, CD16, CD16

## Abstract

**Purpose:**

To evaluate the efficacy of the use of oral Prednisolone and heparin versus the use of heparin alone in treatment of patients with unexplained recurrent miscarriage.

**Methodology:**

The study was a double-blind placebo randomized control trial conducted on 160 patients with unexplained recurrent miscarriage. Patients recruited were randomized into two groups. The first group received oral Prednisolone in addition to low dose aspirin and heparin, while the other group received a placebo in addition to low dose aspirin and heparin. A peripheral venous blood sample was taken from all included patients before starting treatment and collected in heparinized tubes. Natural Killer (NK) cells were checked in each sample and then re-checked in another sample at 20 weeks of gestation.

**Results:**

We found that in the prednisolone group, 70.3 % of women had successful outcome (defined as an ongoing pregnancy beyond 20 weeks gestation), while 29.7 % miscarried before this gestation. On the contrary, among women in the placebo group, 9.2 % had successful outcome while 90.8 % miscarried before 20 weeks, which was statistically significant. On the other hand, we found that there were no significant paired differences between initial serum levels of the NK cells markers CD16 and CD56 and their levels at 20 weeks gestation in both groups.

**Conclusion:**

The addition of prednisolone to heparin and low dose aspirin might be beneficial in patients with unexplained recurrent miscarriage, and this effect might be due to a suppressive effect of steroids on the peripheral CD16 NK cells concentration.

## Introduction

Recurrent miscarriage (RM) may be defined as two or more failed pregnancies (confirmed by ultrasonography or histopathological examination) and is known to affect approximately 0.5–1 % of couples [1].

Various maternal and foetal factors are implicated in the pathophysiology of RM. Genetic abnormalities with chromosomal, single gene, or genomic imprinting defects account for 3.5–5 % of the causes of RM. [2] Other foetal defects include foetal infections and developmental abnormalities [3]. Maternal causes of RM include immunological causes accounting for 30 % of the cases, with anti phospholipid antibody syndrome being the most common autoimmune cause [4, 6]. Endocrine dysfunction accounts for 48.71 % of the causes [5], while other maternal factors including anatomical detects and sub-clinical endometrial infection account for a minimal number of cases [7–9]. Unfortunately, approximately 50 % of the RM cases are unexplained with no definitive aetiology. Several authors are proposing the cause to be alloimmune rejection of the foetus [10].

Natural killer (NK) cells are lymphocytes that are part of the innate immune system and are found in both peripheral blood and uterine mucosa [11, 12]. It has been found that in women with a history of RM, 37.3 % had a mild to moderate increase (12–18 %) and 14.7 % a marked increase (>18 %) in peripheral blood CD56^+^ NK cells. An NK cell percentage of <12 % is strongly associated with a subsequent successful pregnancy [20, 26]. However, these findings have not been confirmed by other studies that concluded that peripheral NK cell numbers do not necessarily correlate with NK cell cytotoxicity, which can be represented by markers such as KIRs (killer inhibitory receptors) or CD16 receptor expression [27].

Up until now, therapy has been empirical and not evidence based, resulting in a poor understanding of the precise aetiology of unexplained RM. Treatments have included aspirin, heparin, progesterone, steroids, leukocyte immunization and IVIG [13].

Heparin and aspirin have been recommended as a standard empiric treatment for cases of RM by the American College of Obstetrics and Gynecology and the Royal College of Obstetrics and Gynecology protocols [14, 15].

Prednisolone has been reported to improve in vitro fertilization treatment outcome and to reduce the rate of miscarriage due to various reproductive immunological problems by increasing the T-regulatory cells, which in turn regulate the peripheral NK cells and their homing into the decidua [16].

The aim of this study was to evaluate the efficacy of adding oral prednisolone to heparin and low dose aspirin in the management of patients with unexplained RM.

## Subjects and methods

This randomized double-blind placebo controlled clinical trial was conducted at Ain Shams University Maternity Hospital and involved 180 patients who experienced unexplained recurrent miscarriage between August 2010 and May 2012.

The sample size was calculated using Epi Info Q version 6.0. Data from a previous study by Thum et al. [17] indicated that a minimum sample size of 76 women in each group was required for an *α* level of 0.05. The total sample size was with an assumed dropout rate of 5 %. The 180 patients were then randomised into two groups.

The patients recruited in the study were aged 18–35 years, with viable current early pregnancy (<7 weeks gestation) and a history of unexplained RM (which was defined as ≥2 previous miscarriages at <20 weeks^,^ gestation) [6].

Patients excluded from the study were those with documented endocrinopathies, uterine anomalies, anti-phospholipid antibody syndrome, thrombophilia, abnormal karyotype in one or both parents, autoimmune disorders, a contraindication to steroid study, a history of hormonal contraception, or a history of intrauterine contraceptive device (IUCD) application within the 3 months preceding the current pregnancy.

The enrolled Patients were randomised into two groups,

- Group I (prednisolone group): patients in this group received oral prednisolone 5 mg/day in addition to the empiric therapy.
- Group II (control group): patients in this group received only the empirical treatment in the form of low- dose aspirin 81 mg/day and unfractionated heparin 5,000 IU subcutaneously injected/12 h.
- Allocation was randomised using dark, sealed envelopes detailing the intervention, which were selected from a table of numbers created by a third party not involved in the allocation process.
- For each patient, an envelope was selected from the sequentially numbered envelopes on the day of recruitment by a nurse not involved in the study.

Independent ethics approval was obtained from the local research ethics committee at Ain Shams University, and written informed consent was obtained from patients willing to participate in the study.

A complete history was obtained, thorough examination, and obstetric ultrasonography were conducted to assess the viability of pregnancy and gestational age for all participants.

A peripheral venous blood sample was taken from all participants before the start of treatment for assessment of NK cells using flow cytometry within 24 h of collection.

NK cells were rechecked in patients who successfully reached 20 week gestation.

The NK cell assay was performed as described below:

Reagents used:

- Phosphate-buffered saline (PBS) (0.8 g/L NACL, 0.2 g/L KCL, 1.15 g/L NaH_2_ PO_4_) added to 100 mL of distilled water with pH adjusted to 7.3 ± 0.2.
- Lysing solution (1.5 mmol/L NH_4_CL, 100 nmol/L KHCO_3_ and 10 mmol/L Na-EDTA) made up to 1 L with distilled water, pH adjusted to 7.2.
- Negative isotype control for identifying the non-specific binding of monoclonal antibodies (MoAb).
- Specific fluorochrome conjugated MoAb against the measured antigen (CD56–CD16).

### Procedure

- In cases of leukocytosis, the blood was diluted with PBS so that the total leukocyte count was 5–10 × 10^3^/mm^3^.
- For each sample, two tubes were prepared, one labelled with the MoAbs used and the other tube the control MoAb.
- Each tube was also labelled with the patient name and date of request, and was processed within 6 h of collection.
- A volume of 50 μL of sample was delivered per each tube.
- A volume of 5 μL of selected MoAb and the control MoAb was added to the respective tubes.
- The tubes were vortexed and incubated in the dark at room temperature for 15 min.
- 1.5 mL of lysing solution was added to each tube.
- The tubes were vortexed again and incubated in the dark at room temperature for 5–10 min.
- Following this incubation, the tubes were centrifuged at 5,000 rpm for 5 min and the supernatant was discarded.
- PBS (2 mL) was added as a wash buffer to each tube and mixed thoroughly.
- The tubes were centrifuged at 3,000 rpm for 3 min and the supernatant was discarded.
- Cells were suspended in 500 μL of PBS to be ready for acquiring data by the flow cytometry.
- The reagent used for flow cytometry was carboxyfluorescein conjugated mouse monoclonal anti-human CD56 and CD16, supplied as 25 μg of antibody in 1 mL PBS containing 0.1 % sodium azide. The isotype was mouse IgG2a.
- The above reagent was designed to quantitatively determine the percentage of cells bearing CD56 and CD16 markers within a population of cells.
- The principle: the washed cells were incubated with the fluorescein- labelled monoclonal antibody, which bound the cells expressing CD56 and CD16. Unbound fluorescein-conjugated antibody was then washed from the cells. Cells expressing CD56 and CD16 were fluorescently stained, with the intensity of staining being directly proportional to the density of expression of the markers. Cell surface expression of the markers was determined by flow cytometry using 488 nm wavelength laser excitation and by monitoring emitted fluorescence with a detector optimised to collect peak emissions at 515–545 nm. Analysis was performed using a Coulter XL Flow cytometer using XL software (Coulter^®^ Epics^®^ XL ™ flow cytometer at Ain Shams University-Hematology Department-Flow Cytometry Laboratory).
- The results were represented as a two-parameter histogram with the *x*-axis representing the CD56 percentage expression and the *y*-axis representing the CD16 percentage expression.

Drugs used as treatment options for both groups were:

- Low-dose aspirin (Juspirin^®^, Julphar, Egypt) was administered at a daily dose of 81 mg/day.
- Heparin was given as unfractionated calcium heparin (Cal-heparine^®^, Amoun, Egypt) at a daily dose of 5,000 IU subcutaneously every 12 h.
- Group I patients received prednisolone tablets (Hostacortin^®^, Sanofi-Aventis, Egypt) at a daily oral single dose of 20 mg/day.
- Group II patients received specially manufactured tablets, resembling Hostacortin^®^ tablets in shape, colour and size. These tablets were manufactured in our pharmacology department.
- Data were recorded on “case record form” from a template by a non-medical personnel.
- Both patients and managing physicians were blinded and group assignment was conducted by a nurse who was not involved in the study.
- Statistical analysis was performed on a personal computer using Microsoft © office 2007 (Excel) and the statistical package for social sciences version 16.0 (SPSS © v. 16.0, SPSS Inc., Chicago, IL, USA).
- A one-sample kolmogorov–Smirnov test was done to test the hypothesis that the variables were normally distributed.
- Normally distributed continuous or discrete data were presented as mean (± standard deviation), and between-group differences were tested parametrically using the independent sample Student’s *t* test.
- Non-normally distributed or discrete data were presented as median (interquartile range) and inter–group differences were tested non-parametrically using the Mann–Whitney *U* test.
- Nominal and categorical data were presented as number (%), and inter-group differences were compared using Pearson’s *χ* ^2^ test with application of Fisher’s exact test when appropriate.
- The relation between the two studied parameters was determined using correlation coefficients.
- A receiver operator characteristic curve was constructed to examine the predictive value for a particular test.
- In all instances, a *P* value > 0.05 was considered non-significant, while *P* value < 0.05 is considered significant.

## Results

This study recruited 180 patients with a history of RM. Twenty patients were excluded owing to declined randomisation or incomplete investigations. The remaining 160 patients were randomised into two groups: 80 in the prednisolone group and 80 in the empirical treatment group. Ten patients were lost to follow up in both groups.

**Fig. 1:**
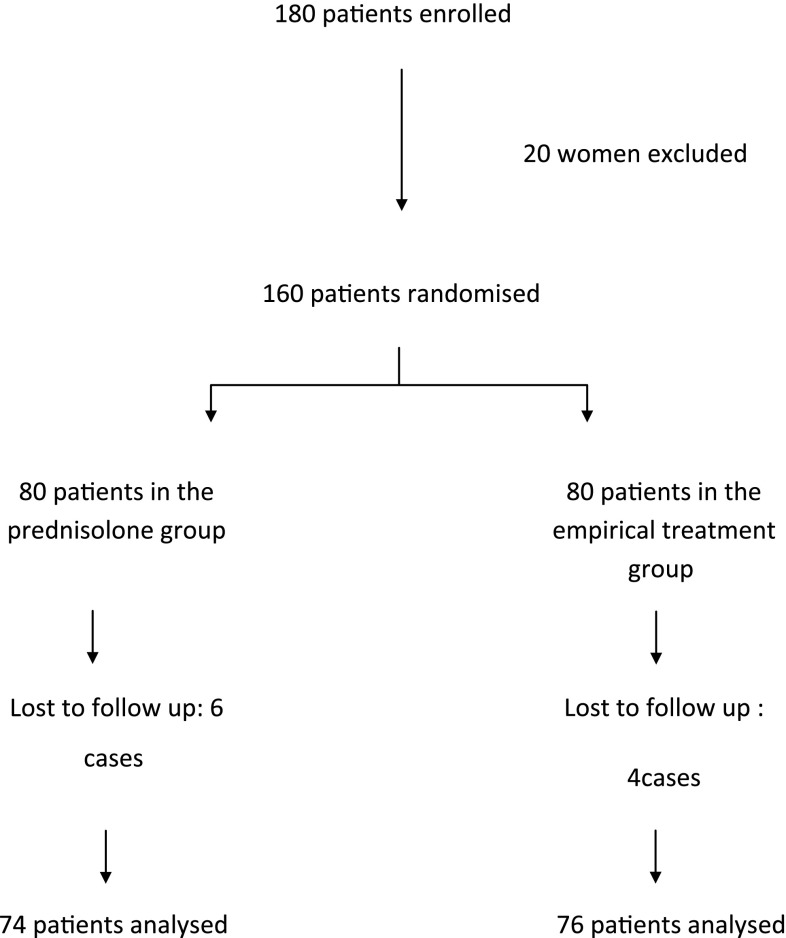
Participant flow in the study

The flow of participants in the study is shown in Fig. 1. There were no significant differences between group I (prednisolone group) and group II (empirical treatment group) patients regarding age, weight, parity and number of previous miscarriages as shown in Table 1.

**Table 1:** Clinical characteristics of patients

	Age (years)		Weight		Parity		Number of previous miscarriages	
	Range	Mean ± SD	Range	Mean ± SD	Range	Median (IQR)	Range	Median (IQR)
Group I	19–34	26.13 ± 4.26	55–99	79.85 ± 11.03	0–2	0 (0–0)	2–10	5 (4–7)
Group II	21–37	27.09 ± 4.09	55–93	77.93 ± 11.24	0–2	0 (0–0)	2–11	6 (4–7)
*P* value	0.147		0.276		0.667		0.488	

*IQR* interquartile range

In the study, we found that 70.3 % of women of Group I had a successful pregnancy (defined as an ongoing pregnancy beyond 20 weeks gestation), whereas 29.7 % miscarried before this gestation. Conversely, among Group II women (empirical treatment group), 9.2 % had a successful pregnancies, whereas 90.8 % miscarried before 20 weeks gestation, constituting a statistically significant difference (an almost eightfold higher rate of successful outcomes and a 61.1 % reduction in the risk of miscarriage before 20 weeks) and a number need-to-treat (NNT) of 1.63 (Table 2).

**Table 2:** Comparison of results between the groups

	Group I (*n* = 74)	Group II (*n* = 76)	Relative risk (95 % CI)	Number needed to treat
Successful outcome	52 (70.3 %)	7 (9.2 %)	7.63 (3.7–15.7)	1.63
Miscarriage	22 (29.7 %)	69 (90.8 %)		

In the study, we also found that the difference between initial serum levels and CD16 serum levels at 20 weeks gestation was significantly higher in women of group I (Prednisolone group) than group II women (empirical treatment group). Conversely, the difference between initial CD56 serum levels and CD56 serum levels at 20 weeks gestation was not statistically significant (Table 3).

**Table 3:** Differences between the groups, regarding differences between the initial and 20 week percentages of CD16 and CD56 in the serum

	Group I (prednisolone treatment) (*n* = 52)	Group II (empirical treatment) (*n* = 7)	*P* value
Difference between initial and 20 week gestation CD16 serum levels			
Range	−5.2 to 17.2	−1 to 0.2	0.008
Median (IQR)	0.3 (0–0.63)	−0.4 (−1 to 0.1)	
Difference between initial and 20 week gestation CD56 serum levels			
Range	−3.06 to 12.7	−2 to 1	0.468
Median (IQR)	0.24 (0–0.71)	0 (−2 to 1)	

Another finding in our study was that there was no significant association between successful outcomes and either initial CD56 or initial CD16 serum levels (Table 4).

**Table 4:** Association between percentage of CD56 and CD16 in the serum and successful outcome

	Odds ratio (95 % CI)	*P* value
Initial serum CD16	1.225 % (1.065–1.0410)	0.085
Initial serum CD56	1.102 % (0.994–1.222)	0.064

## Discussion

A successful pregnancy necessitates adaptation of the maternal immune response to the semi-allogeneic developing embryo, and as NK cells are part of the maternal innate immune system, it has been postulated that NK cells may play a role in the maintenance of pregnancy [11–23].

NK cells are known to be found in both peripheral blood and the endometrium. Although both peripheral NK cells (pNK) and uterine NK cells (uNK) express the surface antigen CD56, they are phenotypically and functionally different [11]. Studies have shown that 90 % of pNK cells express a CD56^dim^ CD16^+^ phenotype, while 80 % of the uNK cells express a CD56^bright^ CD16^−^ phenotype, with the CD56 cells known to have a regulatory function, while the CD16 cells have a cytotoxic function [11–21]. In humans, it has been proved that the elevated levels of circulating cytotoxic NK cells—and not the absolute count of NK cells—increase the risk of miscarriage [27].

In this study, we tried to determine the efficacy of steroid therapy in patients with unexplained RM and the effect of steroid therapy on both the count of pNk cells and their cytotoxic function.

We found that oral prednisolone was associated with an eightfold increase in successful on-going pregnancies beyond 20 weeks gestation [(RR 7.63, 95 % confidence interval (3.71–15.7)].

Oral Prednisolone was also associated with a 61.1 % reduction in the risk of miscarriage before 20 weeks gestation (NNT = 1.63). Although a different design was used, in their study, Lash and his colleagues found that administration of Prednisolone pre-pregnancy helped one-third of their participants to achieve a live birth [18]. But a recent study published by Tang et al., on the use of prednisolone in patients with high levels of uterine natural killer (uNK) cells, stated that live birth rate among the prednisolone group was 60 % and among the placebo group it was 40 % with a risk ratio of 1.5, and hence was not significant. The discrepancy between our results and theirs may be due to the insufficient sample size and the inconsistency in the start date of treatment, as they stated in their conclusion [28].

One of the beneficial effects of prednisolone might be its suppressive effect on NK cells. In our study, we found that prednisolone effectively suppressed peripheral CD16 levels in women with a successful pregnancy until 20 weeks gestation; however, this suppressive effect was not found in the levels of CD56 cells. These findings agree with the speculations mentioned above that suppression of the cytotoxic function of NK cells by suppressing the CD16 expressing cells may have a beneficial effect on patients with RM. Again, this was consistent with the findings of Lash et al. [18], which found that prednisolone reduced uNK cells in the majority of their cases.

In our study, we failed to find an association between pNK cell (CD56 CD16) levels before the start of any treatment and the miscarriage rate, which indicates that such cells can’t be used as predictors of a successful pregnancy outcome. Although different study designs have been used among study groups, the results of two studies concur with our speculations and the finding that pNK cell levels cannot predict the risk of spontaneous abortion in women with unexplained RM [19, 20].

It is worthy of mention that in our study we found that in the non-prednisolone group (the group receiving only low dose aspirin and unfractionated heparin), the ongoing pregnancy rate was only 9.2 %, which contradicts the findings of Badawy et al. [24] and Fawzy et al. [25], whose studies were also conducted in Egypt. There may be two reasons for this difference. First, the authors of these studies used low molecular weight heparin enoxaparin 20 mg/day subcutaneously, whereas we used unfractionated heparin in our study for financial reasons. Second, in our study, the timing of the start of therapy differed from theirs. In their study, they began therapy as soon as foetal life was documented, which was not the case in our study [24, 25].

Our study has limitations in that we did not follow the pregnancies up to delivery, and heterogeneity existed in the start time of therapy, which might have affected the outcome in some cases.

In conclusion, this randomized, double-blind placebo controlled trial showed that prednisolone therapy has a beneficial effect when combined with heparin and low dose aspirin in the management of patients with unexplained RM, which might be due to the suppressive effect of steroids on the cytotoxic CD16 cells.
